# Gut microbiota alterations are distinct for primary colorectal cancer and hepatocellular carcinoma

**DOI:** 10.1007/s13238-020-00748-0

**Published:** 2020-08-14

**Authors:** Wei Jia, Cynthia Rajani, Hongxi Xu, Xiaojiao Zheng

**Affiliations:** 1 Center for Translational Medicine and Shanghai Key Laboratory of Diabetes Mellitus, Shanghai Jiao Tong University Affiliated Sixth People’s Hospital, Shanghai, 200233 China; 2 Hong Kong Tranditional Chinese Medicine Phenome Research Center, School of Chinese Medicine, Hong Kong Baptist University, Kowloon Tong, 999077 Hong Kong, China; 3 University of Hawaii Cancer Center, Honolulu, HI 96813 USA; 4 School of Pharmacy, Shanghai University of Traditional Chinese Medicine, Shanghai, 201203 China

**Keywords:** gut microbiota, colorectal cancer, hepatocellular carcinoma

## Abstract

Colorectal cancer (CRC) and hepatocellular carcinoma (HCC) are the second and third most common causes of death by cancer, respectively. The etiologies of the two cancers are either infectious insult or due to chronic use of alcohol, smoking, diet, obesity and diabetes. Pathological changes in the composition of the gut microbiota that lead to intestinal inflammation are a common factor for both HCC and CRC. However, the gut microbiota of the cancer patient evolves with disease pathogenesis in unique ways that are affected by etiologies and environmental factors. In this review, we examine the changes that occur in the composition of the gut microbiota across the stages of the HCC and CRC. Based on the idea that the gut microbiota are an additional “lifeline” and contribute to the tumor microenvironment, we can observe from previously published literature how the microbiota can cause a shift in the balance from normal → inflammation → diminished inflammation from early to later disease stages. This pattern leads to the hypothesis that tumor survival depends on a less pro-inflammatory tumor microenvironment. The differences observed in the gut microbiota composition between different disease etiologies as well as between HCC and CRC suggest that the tumor microenvironment is unique for each case.

## Introduction

Hepatocellular carcinoma (HCC) is the third most common cause of death by cancer with a high mortality rate (Patel et al., 2012). Approximately 50% of HCC are induced by hepatitis B (HBV) infection and 20% by hepatitis C (HCV) infection. Viral infection acts as a first hit to produce liver inflammation that can develop into cirrhosis over time, and approximately 70%–90% of HCC cases occur in conjunction with cirrhosis (Guo et al., 2018). CRC is the second most common cause of death from cancer and has high incidence in Western countries. The incidence is currently increasing in Asian countries, and environmental factors such as diet have a large impact (Park et al., 2018). Excessive consumption of red meat and high fat influences the composition of the gut microbiota which in turn, can produce metabolites that contribute to intestinal inflammation resulting in the initial carcinogenic milieu for CRC (Feng et al., 2015). Pathological changes in the gut microbiota, referred to as “gut dysbiosis”, that lead to inflammation in the intestine is a common feature of both CRC and HCC. However, primary CRC and HCC develop as distinctive tumors in the intestine and liver, respectively. The connection between gut microbiota composition and both CRC and HCC has been well studied in animal models (Xie et al., 2016b; Wong et al., 2017). There are three categories of gut dysbiosis: 1) loss of beneficial, commensal bacteria, 2) enhanced abundance of pathobionts, and 3) loss of overall microbial diversity. These categories often occur concurrently (Petersen and Round, 2014).

## HCC and CRC Development

Tumors are composed of a variety of cells such as fibroblasts, leukocytes, endothelial cells as well as cancer cells which together comprise the tumor micro-environment (TME). Within this TME, different cell types can signal to one another and recent studies have suggested that it is the cancer cells that are the “organizers” of the TME and are the principle source of the cellular signaling responsible for the induction and final formation of the TME (Li and Stanger, 2019). In the liver, cells such as hepatic stellate cells (HSC), Kupfer cells (liver macrophages, KC), pit cells, dendritic cells, natural killer T-cells (NKT) along with hepatic sinusoid endothelial cells (LSEC) form the surrounding environment or stroma for hepatocytes/HCC tumor cells (Ohtani and Kawada, 2019).

The path to HCC involves several stages which are the results of chronic hepatocyte death, inflammation and repair of liver tissue (Wu et al., 2014b). In non-viral HCC, the gut microbiota can gain access to the liver as result of a chronic liver disease (CLD) associated dysfunction of the intestinal barrier that is the result of high-fat diet (HFD), alcohol, and/or increased amounts of secondary bile acids (BAs) such as deoxycholic acid (DCA) (Ohtani and Kawada, 2019). The “leaky” intestinal membrane allows for translocation of bacteria-derived LPS (gram-negative bacteria) and lipoteichoic acid (LTA, derived from gram-positive bacteria) which bind to and activate the toll-like receptor-4 (TLR-4). Subsequently, the activation of TLR-4 by LPS and/or LTA initiates the nuclear factor-kappa-B (NF-κB) inflammatory signaling pathway and ultimately leads to production of the inflammatory cytokines IL-1β and IL-18 (Loo et al., 2017). HCC with cirrhosis or non-alcoholic steatohepatitis (NASH) has often been associated with continuous LPS/LTA-TLR-4 signaling activation. Increased levels of gut microbiota derived DCA has been associated with HCC with NASH (Stenman et al., 2013; Loo et al., 2017). The inflammatory pathway JNK/p38 → NF-κB → IL-18/1β → ↑HSC activation, ROS →↑fibrosis, can be activated not only by the lipotoxicity derived from excess hepatic lipid storage but also from DNA damage caused by DCA in NASH (Bernstein et al., 2005; Ferreira et al., 2014; Chow et al., 2017; Ohtani and Kawada, 2019). NASH is also closely related to mitochondrial dysfunction as lipid accumulation in hepatocytes causes increased activation of peroxisome proliferator activated receptors (PPARs) leading to elevated ROS, increased oxidative stress, cell death and activation of fibrogenic HSCs (George et al., 2003). Mitochondrial derived ROS can also induce production of the inflammatory cytokines, TGF-β, TNF-α and IL-6 (Chen et al., 2019). Another gut microbiota derived metabolite associated with NASH and liver inflammation is trimethylamine (TMA) which is derived from choline metabolism and can be converted in the liver to a highly toxic and pro-inflammatory compound, trimethylamine oxide (TMAO) which not only causes hepatocyte damage but also results in choline deficiency and progression of hepatic steatosis with increased HCC risk (Chu et al., 2019).

Virus-associated HCC begins with an acute immunogenic insult due to infection with either hepatitis B or C virus (HBV or HCV). First, there is necrosis of hepatocytes due to the viral infection which activates the HSCs through the paracrine action of cytokines released by the necrotic hepatocytes. Second, HSCs are further stimulated by infiltrating activated KC cells and leukocytes to evolve into fibrogenic myofibroblasts which in turn release cytokines to stimulate over-production of ECM components including collagens I, III, laminin and fibronectin that result in an imbalance of ECM production and degradation which is termed fibrosis (Wu et al., 2014b; Ohtani and Kawada, 2019). HSCs also contribute to the development of the TME. An activated HSC can undergo senescence and become a senescence-associated secretory phenotype (SASP) that secretes tumor progression promoting cytokines (IL-6, IL-8), chemokines and proteases (matrix metalloproteinases, MMPs) which act to remodel the ECM and enhance inflammation (Rodier and Campisi, 2011; Rao and Jackson, 2016).

**Figure 1:**
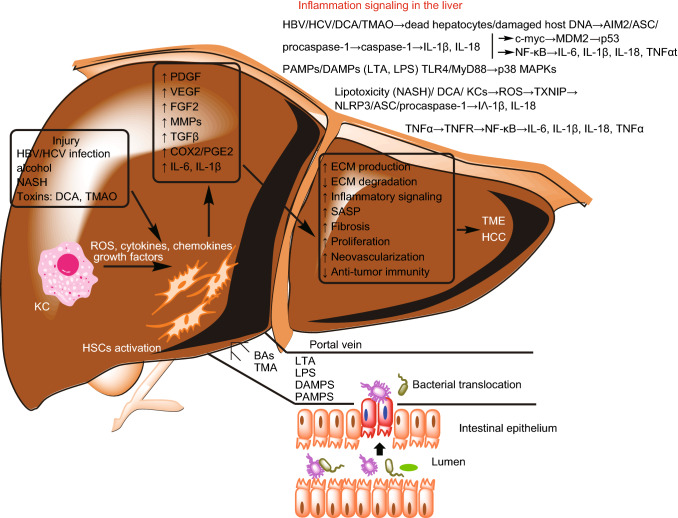
**Tumor formation in HCC**. CLD → HCC starts with injury to the liver usually by viral infection with either HBV or HCV virus, or exposure to toxins such as TMAO and DCA (secondary BAs). The initial injury causes hepatocyte death with subsequent activation of KCs and HSCs that release pro-inflammatory substances and initiate a vicious cycle of liver damage and repair. The gut microbiota also produces substances such as LTA and LPS which are capable of activating TLR4 inflammatory pathways in the liver. HSC and KC activation lead to increased production of growth factors, PDGF, VEGF and FGF2 which contribute to the TME by enhancing hepatocyte proliferation and neovascularization. Increased production of TGFβ and MMPs acts together to increase ECM production while increased COX2 and PGE2 levels modulate the immune system in such a way as to create an immunosuppressive environment to protect the HCC tumor. The major inflammatory pathways that contribute to increased fibrosis/cirrhosis are written out in the top right of the figure.

KCs are resident liver macrophages that perform phagocytosis to remove microbial debris in the form of damage-associated molecular patterns (DAMPs) primarily derived from damaged hepatocytes, microbial associated patterns (MAMPs) such as lipopolysaccharides (LPS), and bacteria from the liver blood flow. When activated by inflammatory cytokines, they secrete further inflammatory cytokines and chemokines to influence activation of HSCs and modulate the immune response by facilitating infiltrations of other types of leukocytes (Krenkel and Tacke, 2017; Ohtani and Kawada, 2019). Liver macrophages can also be restorative though their immune modulating effect on T-, B-, T- and NK-T cells which act to inactivate HSCs and allow resolution of the inflammation. When an imbalance exists between fibrosis and resolution of the inflammation driving it, increased fibrosis and liver cirrhosis results which often leads to HCC (Krenkel and Tacke, 2017). Figure 1 summarizes the path from inflammation to CLD to HCC.

**Figure 2:**
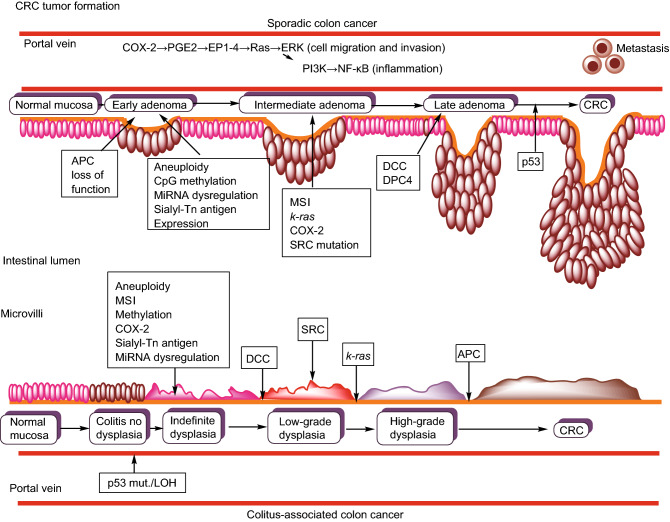
**Tumor formation in sporadic and colitis-associated CRCs**. Sporadic CRC begins with a mutation in the tumor suppressor gene, adenomatous polyposis coli (*APC*). The resulting dysplastic adenomas typically develop in a chronically inflamed mucosa which promotes chromosomal and microsatellite instability, hypermethylation of gene promoter regions and oxidative stress. The adenomas progress sequentially from early (*APC* mutation) → intermediate→ late adenoma→ carcinoma (loss of p53 tumor suppressor gene). Colitis-associated CRC (CCRC) develops dysplastic lesions that are often flat rather than distinct adenoma polyps and the progression to CRC starts with colitis with no dysplasia (loss of p53) → indefinite dysplasia→ low-grade dysplasia → high-grade dysplasia (loss of *APC*) → carcinoma.

CRC, like HCC, develops slowly over more than 10 years in an environment of chronic inflammation and repair. Like HCC, the etiology of CRC varies and this invokes different aberrations from normal intestinal mucosa. The two major types of CRC, sporadic (SCRC) and colitis-associated (CCRC) differ in their histologic presentation and the timing/sequence of cellular mutations (Ullman and Itzkowitz, 2011; Dekker et al., 2019). Figure 2 summarizes the differences/similarities between sporadic and colitis-associated CRC types. Colitis, like CLD is often triggered by environmental insults such as bacterial or viral infections which in turn, activate an immune response that leads to the initial inflammation setting up a cycle of prolonged, repeated ulceration followed by re-epithelialization with increasingly abnormal clones of aneuploid cells (Xavier and Podolsky, 2007). In the intestine, tumor-associated myeloid cells produce IL-23 which affect Th17 cell polarization and the subsequent production of the cytokines IL-17A, IL-21, TNF-α and IL-6 which in turn, provide the pro-tumorigenic inflammatory response in CRC (De Simone et al., 2013; Long et al., 2017). IL-6 activates the signal transducer and activator of transcription-3 (STAT3) signaling pathway which in turn, acts to promote tumor growth (De Simone et al., 2015) and anti-tumoral immunity (Wu et al., 2014a). IL-6 also activates the NF-κB signaling pathway which helps to perpetuate inflammation and promote tumorigenesis (Koliaraki et al., 2015). Both phosphorylated STAT3 (activated) (Lin et al., 2011) and NF-κB (Myant et al., 2013) levels have been reported to be high in tumor-initiating cells, a subpopulation within the bulk tumor with “stem-like” characteristics (Schwitalla et al., 2013; Long et al., 2017).

The intestine is densely populated with microorganisms and several species have been associated with CRC. In general, early stages of CRC have been characterized by a decrease in commensal bacteria such as *Lactobacillus*, *Bifidobacterium* and *Clostridium* which are known to produce anti-inflammatory SCFAs and were negatively correlated with increased markers for damaged intestinal epithelium such as diamine oxide (DAO), D-lactate and LPS. *Fusobacterium nucleatum* and *Escherichia coli* (pks+) were found to be positively correlated with DAO. LPS and D-lactate (Liu et al., 2020).

*Fusobacterium nucleatum* has been shown to interact directly with CRC tumor cells where its adhesin, FadA binds to E-cadherin on the CRC cell surface and activates Wnt/β-catenin signaling (Rubinstein et al., 2013). *F*. *nucleatum* has been shown to bind to the T-cell inhibitory immune receptor of natural killer cells (NK) through another adhesin, Fap2 (Gur et al., 2015). Fap2 has been reported to facilitate increased binding of *F*. *nucleatum* to CRC cells via its ability to bind to a highly expressed CRC cell surface marker, the disaccharide sugar motif [galactose *N*-acetyl-D-galactosamine (Gal-GalNAc)] (Coppenhagen-Glazer et al., 2015). *F*. *nucleatum* has also been shown to activate the cellular survival mechanism, autophagy, through TLR4 receptors located on CRC cell surfaces in response to oxaliplatin chemotherapy (Yu et al., 2017). The enterotoxic bacteria, *Bacteroides fragilis* (ETBF), causing GI inflammation has been reported to inhabit biofilms coating human CRCs and precancerous colonic adenomas (Boleij et al., 2015). Similarly, *Escherichia coli* expressing the genomic island polyketide synthase (pks+), have been detected in human CRC tissues. The genotoxin produced by pks+ *E*. *coli*, colibactin, alkylates the DNA of colonic epithelial cells and is thus a source of potential mutations leading to CRC (Arthur et al., 2012). It must be emphasized that many other bacteria as yet to be well characterized may also play a role in CRC carcinogenesis and progression.

An important topic of continuing research is an understanding of what compositional changes in the microbiota, if any, occur as pathogenesis to the final tumor stage of both CRC and HCC occurs. In this review we will examine several recent studies that highlighted the differences in gut microbiota composition between different disease etiologies and stages for CRC and HCC. The papers discussed in this review were selected using the following criteria: 1) published within the last five years, 2) focused on comparison of at least two stages of disease, 3) for HCC, focused on a single etiology for liver disease, 4) were human studies only, and 5) taxonomy sequencing was done at the genus or species level. Few studies met all of these criteria and the most current of these, with the largest sample sizes, were chosen in this review. The tables in this review listed changes in microbiota genera and also whether the changes would either promote or inhibit inflammation (anti- or pro-). For some cases the change can be either anti- or pro-inflammation depending on the bacterial species or presence of other immune-modulating bacteria. Details are given in the text below the table along with references for each genus listed with pro- or anti-inflammation properties.

## A Comparison of Differential Bacterial Compositions Associated with HCV vs. Healthy Controls

Our discussion of viral induced HCC begins with the examination of the impact on the gut microbiota by the initial “hit” of virus infection. Several studies have suggested that not only the stage of liver disease but also the stage of HCV infection were responsible for changes in the gut microbiota composition (Aly et al., 2016; Heidrich et al., 2018; Inoue et al., 2018). HCV infection progresses through 4 chronological stages: 1) persistently normal serum alanine aminotransferase (PNALT), 2) chronic hepatitis (CH), 3) liver cirrhosis (LC) and 4) HCC (Inoue et al., 2018).

A recent study (Table 1) of the fecal microbiome for six chronic, stage 4 HCV patients with no other underlying disease, and eight healthy controls (HC) from the same geographical region was performed and their results showed that the microbiota richness and diversity of HCs was higher than that of HCV patients (Aly et al., 2016). Lower diversity has been shown by others to indicate chronic inflammation (Lozupone et al., 2012; Chen et al., 2015; Giloteaux et al., 2016).

**Table 1:** Microbiota differences between stage 4 HCV (HCC) patients and healthy controls at the genus level*

Increased in HC	Pro- or anti-inflammation	Increased in HCV	Pro- or anti-inflammation
*Ruminococcus*	Anti-	*Acinetobacter*	Pro-
*Parabacteroides*	Anti-	*Prevotella*	Anti- or pro-
*Butyricimonas*	Anti-	*Veillonella*	Anti- or pro-
*Bifidobacterium*	Anti-	*Phascolarctobacterium*	Anti- or pro-
*Clostridium*	Anti-	*Faecalibacterium*	Anti-
*Lachnospira*	Anti-	*Streptococcus*	Anti- or pro-
*Bacteroides*	Anti- or pro-	*Blautia*	Anti-

*Data from (Aly et al., 2016)

Most of the bacteria enhanced in HCs are plant polysaccharides and fiber fermenters, such as *Ruminococcus*, *Clostridium*, *Lachnospira*, and *Bacteroides*, and may participate in cross-feeding each other, as well as other commensal bacteria (La Reau and Suen, 2018). *Lachnospira*, *Butyricimonas* and *Clostridium* especially those in cluster XIVa and IV are acetic acid and butyric acid-producing bacteria, are anti-inflammatory and promote healthy colonocytes (Sakamoto et al., 2009; Lopetuso et al., 2013; Hibberd et al., 2017). *Bifidobacterium* and *Bacteroides* have bile salt hydrolase allowing them to deconjugute BAs and exert metabolic benefits via BA signaling (Wexler, 2007; O’Callaghan and van Sinderen, 2016). Commensal *Parabacteroides* has recently been found to have metabolic benefits via secondary BA signaling as well as succinate-activated intestinal gluconeogenesis (Wang et al., 2019). Among those bacteria enhanced in HCV-HCC, *Prevotella* and *Phascolarctobacteria* can be a source of LPS as they are gram negative organisms and thus can be pro-inflammatory (Wu et al., 2017; Schwenger et al., 2018). Some reports also showed that these two bacteria could be anti-inflammatory, depending on the microenvironment and their species or strains. Similarly, *Veillonella* can also be either pro-inflammatory or anti-inflammatory. When co-inhabitating the gut with a specific strain of *Streptococcus,* co-stimulation of *Veillonella* with *S*. *salvarius strain 1* led to decreased amounts of inflammatory cytokine production whereas with strain 2, there was a notable increase relative to either microbe alone (van den Bogert et al., 2014).

It appears from this small study that HCV-HCC generates quite a few anti-inflammatory microbes and several that are immune-modulating. This is not surprising because one could hypothesize that as a tumor matures, it needs to acquire homeostasis with its environment in order to survive and thus an anti-inflammatory, immunosuppressive TME is more desirable.

Another cross-sectional study (Table 2) aimed to delineate the effect of hepatitis C viral infection from liver cirrhosis on the gut microbiota. The intestinal microbiota of 95 patients chronically infected with HCV (*n* = 57 without cirrhosis (NCIR); *n* = 38 with cirrhosis (CIR)) and 50 HC were examined. Microbial diversity was significantly decreased from HC to NCIR to CIR (Heidrich et al., 2018). The alpha diversity was found to be according to the rank order, CIR < NCIR < HC.

**Table 2:** Six definite patterns of relative abundances observed for HC, NCIR and CIR groups (genus level)*

Genus	Pattern	HC (%)	NCIR (%)	Anti- or pro-inflammation	CIR (%)	Anti- or pro-inflammation
*Lactobacillus*	1A	0.076	0.627 (8-fold↑)	Anti- or pro-	0.958 (12-fold↑)	Anti- or pro-
*Streptococcus*	1A	0.542	1.427 (3-fold↑)	Anti- or pro-	2.667 (5-fold↑)	Anti- or pro-
*Veillonella*	1A	0.037	0.115 (3-fold↑)	Anti- or pro-	0.283 (8-fold↑)	Anti- or pro-
*Alloprevotella*	1A	0.115	0.445 (4-fold↑)	Pro-	0.830 (7-fold↑)	Pro-
*Bilophila*	1B	0.090	0.053 (1.6-fold↓)	Pro-	0.033 (3-fold↓)	Pro-
*Clostridim IV*	1B	0.589	0.341 (1.7-fold↓)	Pro-	0.178 (3-fold↓)	Pro-
*Clostridium XIVb*	1B	0.398	0.230 (1.7-fold↓)	Pro-	0.119 (3-fold↓)	Pro-
*Mitsuokella*	1B	0.157	0.054 (3-fold↓)	Pro-	0.005 (31-fold↓)	Pro-
*Vampirovibrio*	1B	0.342	0.210 (1.6-fold↓)	Pro-	0.056 (6-fold↓)	Pro-
*Akkermansia*	2A	0.047	0.030 (1.6-fold↓)	Pro-	0.121 (2.6-fold↑)	Anti-
*Bifidobacterium*	2A	0.122	0.095 (1.3-fold↓)	Pro-	0.207 (1.7-fold↑)	Anti-
*Escherichia-Shigella*	2A	0.739	0.958 (1.3-fold↑)	Pro-	2.390 (3.2-fold↑)	Pro-
*Haemophilus*	2A	0.042	0.057 (1.4-fold↑)	Pro-	0.155 (3.7-fold↑)	Pro-
*Micrococcus*	2A	0.010	0.011(NC)	NC	0.034 (3.4-fold↑)	Pro-
*Weissella*	2A	0.000 not detected	0.002 detected	Pro-	0.122 (61-fold↑)over NCIR	Pro-
*Butyricimonas*	2B	0.162	0.156 (1-fold↓)	Pro-	0.080 (2-fold↓)	Pro-
*Victivallis*	2B	0.046	0.041 (1-fold↓)	NC	0.016 (3-fold↓)	NC
*Citrobacter*	3A	0.000 not detected	0.020 detected	Pro-	0.022 (1-fold↑)	Pro-
*Clostridium sensu stricto*	3A	0.197	0.493 (2.5-fold↑)	Anti-	0.425 (2.1-fold↑)	Anti-
*Pediococcus*	3A	0.000 not detected	0.109 detected		0.093 (1.2-fold↓) over NCIR	
*Flavonifractor*	3B	0.134	0.066 (2-fold↓)	Pro-	0.086 (1.6-fold↓)	Pro-
*Megasphaera*	3B	0.134	0.014 (9.6-fold↓)	Pro-	0.017 (7.9-fold↓)	Pro-
*Pseudoflavonifractor*	3B	0.076	0.051 (1.5-fold↓)	Pro-	0.041 (1.8-fold↓)	Pro-
*Acetivi*brio	3B	0.036	0.007 (5-fold↓)	Pro-	0.005 (7.2-fold↓)	Pro-

Pattern 1A/1B: positively/negatively correlate with the stage of fibrosis

Pattern 2A/2B: positively/negatively correlate with the stage of cirrhosis

Pattern 3A/3B: positively/negatively correlate with chronic hepatitis C

*Data obtained from (Heidrich et al., 2018)

An important finding here was that infection with the virus had a unique impact on the composition of the gut microbiota independent of CLD. In pattern 3A, two bacteria are not detectable until the disease is present and one bacterium exhibits a slight increase with CLD present that does not increase with CLD progression to CIR. Pattern 3B is comprised of 4 bacteria that decrease quite a bit in abundance when CLD reaches the fibrosis state. As to fibrosis, Pattern 1A shows increased *Streptococcus* and *Veillonella* which increase even more as CLD progresses to CIR and can be pro-inflammatory or anti-inflammatory depending on the strain of *Streptococcus*. *Lactobacillus* are normally considered beneficial to host health but they produce bile salt hydrolase which can lead to the production of more primary unconjugated BAs that are better activators of FXR and can thus decrease BA production as well as increased secondary BAs such as DCA that may be hepatotoxic (Ridlon et al., 2006; Bernstein et al., 2011). Pattern 1B contains mostly *Clostridium* species and their decrease with disease progression may signify loss of commensal beneficial bacteria. Pattern 2A has two beneficial bacterial genera, *Akkermansia* and *Bifidobacterium* that decrease in abundance in the NCIR but then increase in the CIR state. The die-off in NCIR followed by a resurgence in CIR may be due to a mutation or opportunistic overgrowth of a particular species in either of these two genera and thus taxonomic classification will have be done at the species level in order to fully understand the data.

To summarize this section, we can see that the infection with HCV by itself alters the gut microbiota allowing pro-inflammatory *Citrobacter* to appear and causing loss of *Clostridia spp*., thus setting the stage for bacterial translocation to occur to the liver. In the fibrotic, NCIR stage there is shift to even more genera of *Clostridium* being decreased accompanied by increased amounts of pro-inflammatory genera which further increases the inflammatory environment in both gut and liver. The trend towards a more pro-inflammatory gut continues for CIR.

The last study (Table 3) to be discussed for HCV pursued the idea of looking at different stages of hepatitis C infection and the effect on gut microbiota composition (Inoue et al., 2018). This study involved 23 HC (healthy control), 18 PNALT (persistently normal alanine aminotransferase), 84 CH (chronic hepatitis), 40 LC (liver cirrhosis) and 24 HCC patients. Microbial diversity was decreased in HCC patients and was associated with the clinical severity of the disease; HCC< LC < CH < PNALT << HC.

**Table 3:** Differential bacteria at the genus level associated with the different stages of HCV (fold-changes were approximated from pie charts)*

Genus	PNALT	Anti- or pro- inflammation	CH	Anti- or pro- inflammation	LC	Anti- or pro- inflammation	HCV-HCC	Anti- or pro- inflammation
*Streptococcus*	~8-fold↑	Anti- or pro-	20-fold↑	Anti- or pro-	30-fold↑	Anti- or pro-	50-fold↑	Anti- or pro-
*Fusicatenibacter*	1.5-fold↑	Anti-	Not detected		Not detected		Not detected	
*Bifidobac terium*	2.5-fold↑	Anti-	3.5-fold↑	Anti-	4-fold↑	Anti-	3-fold↑	Anti-
*Parabacteroides*	1.2-fold↑	Anti-		Anti-	1.2-fold↑	Anti-	2-fold↑	Anti-
*Bacter-oides*	1.2-fold↑	Anti- or pro-	1.5-fold↓	Anti- or pro-	2-fold↓	Anti- or pro-	2-fold↓	Pro-
*Prevotella*	2.5-fold↑	Anti- or pro-	4-fold↑	Anti- or pro-	2.5-fold↑	Anti- or pro-	3-fold↑	Anti- or pro-
*Ruminococcus*	2-fold↓	Pro-	2-fold↓	Pro-	2-fold↓	Pro-	3-fold↓	Pro-
*Lachnospiraceae*	NC		1.5-fold↓	Pro-	1.5-fold↓	Pro-	1.5-fold↓	Pro-
*Blautia*	10-fold↓	Pro-	8-fold↓	Pro-	9-fold↓	Pro-	10-fold↓	Pro-
*Anaerostipes*	NC		NC		NC		detected	
*Clostridium*	Not detected		Not detected		Not detected		Not detected	
*Lachnospiraceae incertae sedis*	3-fold↓	Pro-	3-fold↓	Pro-	4-fold↓	Pro-	4-fold↓	Pro-

Fold-change: The relative abundances relative to normal control

*Data taken from (Inoue et al., 2018)

NC means “no change”

When looking at the changes caused by viral infection (PNALT and CH), one can understand how an unfavorable gut microenvironment is developed. Loss of the genera *Clostridium* along with decreased *Ruminococcus* and *Lachnospiraceae*, all dominant commensal bacteria that are short chain fatty acid (SCFA) producing signifies reduced control of inflammation as SCFAs, especially, butyrate regulate the differentiation of T_reg_ cells (Furusawa et al., 2013). SCFAs are also nutrients for colonic epithelial cells and modulate colonic pH and their decrease results in an increased fecal pH and increased levels of ammonia (Wong et al., 2006). The most noteworthy change in this study was the large overgrowth in *Streptococcus*, a urease-producing bacteria, which has been associated with minimal hepatic encephalopathy (MHE), a condition often found in LC patients (Zhang et al., 2013). *Streptococcus* has also been reported to downregulate the innate immune system and as it is most dominant microbe change observed in HCC, may contribute to tumor survival and modeling of the TME (Cosseau et al., 2008). Its increased abundance was also observed in the second study discussed in this section (Heidrich et al., 2018). Another interesting change is the increase and then disappearance of *Fusicatenibacter*, butyrate producing bacteria which are part of *Clostridium cluster IV* (Rapozo et al., 2017). In this study, the inflammation status of the organisms present remains even across the stages of disease based on their fold changes.

## A Comparison of Differential Bacterial Compositions Associated with HBV vs. Healthy Controls

We then examined the impact of HBV infection on the gut microbiota. The first study (Table 4), regarding HBV, focused on early CHB before the onset of liver damage and metabolic disorders in order to determine whether gut dysbiosis occurred prior to and if it contributed to the pathogenesis of liver disease (Wang et al., 2017).

**Table 4:** Changes in gut microbiota composition in early CHB relative to healthy controls (genus level).

Decreased in CHB	Anti- or pro- inflammation	Increased in CHB	Anti- or pro- inflammation
*Alistipes*	Pro-	*Streptococcus*	Anti- or pro-
*Bacteroides*	Anti- or pro-	*Veillonella*	Anti- or pro-
*Parabacteroides*	Pro-	*Haemophilus*	Pro-
*Ruminococcus*	Pro-	*Lachnospiraceae*	Anti- or pro-
*Clostridium IV*	Pro-	*Megamonas*	Anti-
*Butyricimonas*	Pro-	*Clostridium sensu stricto*	Anti-
*Escherichia-Shigella*	Anti-	*Actinomyces*	Pro-

*Data from (Wang et al., 2017). Note: “anti-” or “pro-” refers to the net result of the change in bacteria abundance*

The alterations due to those bacteria which are reduced in CHB patients result in reduced productions of SCFAs and antibacterial peptides while those bacteria which are enhanced in CHB were closely connected to the host’s physical indices and the accumulation of serum metabolites. These included cholesterol, L-aspartic acid, L-tyrosine, L-phenylalanine, octanoic acid and 1-napthol. The accumulation of aromatic amino acids such as L-phenylalanine and L-tyrosine, may affect cerebral functions and play a causal role in hepatic encephalopathy (Dejong et al., 2007). Notice also the “pairing” of *Veillonella* and *Streptococcus* as seen for HCV-HCC. It should be noted that there is increased abundance of *Lachnospiraceae,* a member of the order Clostridia. Certain strains have been associated with the development of diabetes (Kameyama and Itoh, 2014). This highlights the importance of deeper taxonomic classification. *Actinomyces* inhabits the gut and becomes an opportunistic pathogen, especially in immune-compromised patients, causing inflammation (Kameyama and Itoh, 2014). Overall, infection with hepatitis B causes a lot of inflammation and this is reflected in the gut microbiota composition.

In a second study (Table 5), changes in gut microbiota composition for HC (*n* = 15), CHB (*n* = 21), LC (*n* = 25) and HCC (*n* = 21) groupings were determined (Zeng et al., 2020).

**Table 5:** Differential bacteria at the genus level relative to HC for CHB, LC and HCC patients*.

CHB	Anti- or pro- inflammation	LC	Anti- or pro- inflammation	HBV-HCC	Anti- or pro- inflammation
*Bacteroides* 2.2-fold↑	Anti- or pro-	*Bacteroides* 2.1-fold↑	Anti- or pro-	*Bacteroides* 2.6-fold↑	Anti- or pro-
*Faecalibacterium* 2-fold↓	Pro-	*Faecalibacterium* 1.8-fold↓	Pro-	*Faecalibacterium* 1.2-fold↓	Pro-
*Streptococcus* 1.2-fold↑	Anti- or pro-	*Streptococcus* 6-fold↑	Anti- or pro-	*Streptococcus* 3.8-fold↑	Anti- or pro-
*Bifidobacterium* 5-fold↓	Pro-	*Bifidobacterium* 8-fold↓	Pro-	*Bifidobacterium* 1.7-fold↓	Pro-
*Roseburia* 2-fold↓(pro-)	Pro-	*Roseburia* 3-fold↓	Pro-	*Rosebura* 5-fold↓	Pro-
*Blautia* NC	NC	*Blautia* 2-fold↓	Pro-	*Blautia* 1.3-fold↓	Pro-
*Ruminococcus* 4-fold↓	Pro-	*Ruminococcus* 4-fold↓	Pro-	*Ruminococcus* 8-fold↓	Pro-
*Dorea* 2-fold↑	Pro-	*Dorea* NC	NC	*Dorea* 4-fold↑	Pro-
*Prevotella* 3-fold↑	Anti- or pro-	*Prevotella* 3-fold↑	Anti- or pro-	*Prevotella* 4-fold↓	Anti- or pro-
*Coprococcus* 2.5-fold↑	Pro-	*Coprococcus* 2-fold ↑	Pro-	*Coprococcus* 1.2-fold↓	Pro-
*Clostridium* 10-fold↓	Pro-	*Clostridium* 10-fold↓	Pro-	*Clostridium* 20-fold↓	Pro-
*Akkermansia* Not detected	Pro-	*Akkermansia* strongly detected	Anti-	*Akkermansia* Not detected	Pro-
*Eubacterium* NC	NC	*Eubacterium* NC	NC	*Eubacterium* Not detected	
*Klebsiella* 1.5-fold↓	Pro-	*Klebsiella* 4-fold↓	Pro-	*Klebsiella* 4-fold↓	Pro-
*Haemophilus* 4-fold↓(anti)	Anti-	*Haemophilus* 2-fold↓	Anti-	*Haemophilus* 3-fold↓	Anti-
*Parabacteroides* detected	Anti-	*Parabacteroides* 2-fold↑	anti	*Parabacteroides* 2-fold↑	Anti-
*Collinsella* Not detected ND	Anti-	*Collinsella* Not detected	Anti-	*Collinsella* Not detected	Anti-
*Catenibacterium* Not detected ND		*Catenibacterium* Not detected		*Catenibacterium* Not detected	
*Veillonella* detected	Anti- or pro-	*Veillonella* 4-fold↑	Anti- or pro-	*Veillonella* 4-fold↑	Anti or pro-

Fold-changes are estimated from bar graphs

*Data taken from (Zeng et al., 2020)

The findings that are different here from previous studies are: 1) decrease in *Bifidobacterium* for all stages but less of a decrease seen for HCC, 2) the occurrence of another pro-inflammatory bacteria, *Dorea* which increases with stage of CLD (Leclercq et al., 2014), 3) the appearance of increased *Coprococcus*, a pro-inflammatory bacteria associated with western HFD which increases in CHB and LC but decreases in abundance in HCC (Pedersen et al., 2019), and 4) the decrease in *Roseburia*, an organism that cross-feeds other beneficial bacteria via it ability to degrade dietary β-mannans, with increasing disease stage (La Rosa et al., 2019).

## Changes in Gut Microbiota Composition Associated with HBV and Non-hepatitis Virus Related HCC

As mentioned above, more than 70% of HCC originates from an initial “hit” from hepatitis virus (Guo et al., 2018). A recent study (Table 6) examined the changes in composition of the gut microbiota associated with hepatitis virus induced HCC vs. non-virus induced HCC (Liu et al., 2019). Both etiological routes to HCC, virus vs. non-virus, ultimately led to liver inflammation and cirrhosis but the changes in gut microbiota were found to be different.

**Table 6:** Differential bacteria (top 35 in abundance) associated with HBV vs. non-viral induced HCC relative to healthy controls at the genus level*.

HBV-HCC	Anti- or pro- inflammation	Non-viral HCC	Anti- or pro- inflammation
*Escherichia-Shigella* 1-fold↓	Anti-	*Escherichia-Shigella* 3-fold ↑	Pro-
*Enterococcus* 1-fold↓	Anti-	*Enterococcus* 1-fold ↑	Pro-
		*Proteus* 1-fold↑	Pro-
*Fusobacterium* 1-fold↓	Pro-	*Fusobacterium* 1-fold↓	Pro-
*Bifidobacterium* 2-fold↑	Anti-	*Bifidobacterium* 1-fold↑	Anti-
*Ruminiclostridium* 1-fold↑	Anti-	*Ruminiclostridium* 1-fold↓	Pro-
		*Lachnoclostridium* 1-fold↓	Pro-
*Phascolarctobacterium* 1-fold↑	Anti-	*Phascolarctobacterium* 1-fold↓	Pro-
		*Subdoligranulum* 1-fold↑	Anti-
*Veillonella* 1-fold↑	Anti- or pro-	*Veillonella* 1-fold↑	Anti- or pro-
*Faecalibacterium* 1-fold↑	Anti-	*Faecalibacterium* 1-fold↓	Pro-
		*Pseudobutyrivibro* 1-fold*↓*	Pro-
		*Roseburia* 1-fold↑	Anti-
*Alloprevotella* 2-fold↑	Pro-	*Alloprevotella* 1-fold↓	Anti-
*Prevotella* 1-fold↑	Anti- or pro-	*Prevotella* 1-fold↓	Pro- or anti-
*Bacteroides* 1-fold↓	Pro-	*Bacteroides* 1-fold↓	Pro-
*Parabacteroides* 1-fold↓	Pro-	*Parabacteroides* 1-fold↑	Anti-
		*Alistipes* 1-fold ↑	Anti-

*Data taken from (Liu et al., 2020)

Several remarkable differences between bacterial relative abundances related to viral-induced HCC relative to non-viral induced HCC are shown in Table 6. The pro-inflammatory, endotoxin-producing *Escherichia-Shigella*, *Enterococcus* and *Proteus* are increased in non-viral HCC but reduced in HBV-HCC. There are more members of the phylum Firmicutes that are decreased in the non-viral HCC group which contributes to a more pro-inflammatory state. Overall, the non-viral-HCC appears to have a more pro-inflammatory profile relative to HBV-HCC indicating perhaps a slightly different TME for non-viral HCC. This may be because the non-viral-HCC begins on an already chronically inflamed infrastructure whereas viral-HCC starts with a dramatic immunogenic response.

In the last study (Table 7) to be discussed regarding HCC in mouse models, we are going to look at the association of BA composition with various stages of non-viral liver disease (Xie et al., 2016a). Examining the shifts in BA metabolism is another approach for assessing the impact the gut microbiota on the modeling of the TME. This is the only mouse centered study that was chosen because it was one of a kind in both its approach to study liver disease stages as well as having gut microbiota data that was sequenced to the genus level. In this study, mice were induced to develop HCC using a combination of HFD and a tumor inducer, streptozotocin. Samples were taken at each stage, steatosis, fibrosis, cirrhosis and HCC and bile acids (BAs) were analyzed at each stage both in liver and feces (Xie et al., 2016a).

**Table 7:** Association of gut microbiota abundances in STZ-HFD-HCC mice relative to HC along with associated BAs in liver and feces*.

Genus	Steatosis	Fibrosis	Cirrhosis	HCC	Liver BAs (HCC)	Fecal BAs (HCC)
*Clostridium*	18↑ anti#	30↑ anti	2.5↑anti	10↑ anti		
*Bacteroides*	5↑anti	4.4↑ anti	3.5↑ anti	6.6↑ anti	↑DCA↑TLCA↑TCDCA pro	
*Desulfovibrio*	18↑pro	18↑ pro	5↑pro	10↑pro	↓DCA↓GCA anti	
*Atopobium*	20 ↑pro	39↑pro	16↑pro	10↑pro	slight ↑TUDCA↓DCA anti	
*Parasutterella*	7↓ anti	6↓ anti	7↓ anti	6↓ anti	↓TLCA↓TUDCA↓TCDCA slight↓TDCA, TCA anti	
*Akkermansia*	20↓pro	9↓ pro	2.4↓ pro	5↓ pro	slight↓DCA, GCA, TDCA, TCA slight ↑TUDCA, TCDCA	
*Odoribacter*	Neg.*	Neg.	Neg.	Neg.	↓TCDCA anti	↓LCA
*Sarcina*	Pos.	Pos.	Pos.	Neg.	↑TCA pro	
*Allobaculum*	Neg.	Pos.	Pos.	Pos.		↓TCDCA, GCA, TUDCA,TLCA, TCA
*Escherichia*	NC	NC	NC	Pos.	↑TCDCA, TLCA pro	
*Subdolgranulum*	Pos.	Pos.	Pos.	Neg.	↑TUDCA, TCA, TDCA, GCA pro	
*Barnsiella*	Neg.	Neg.	Neg.	Neg.	↓TCDCA, TUDCA, TLCA anti	↓LCA
*Paraprevotella*	Neg.	Neg.	Neg.	Neg.	↓TCDCA, TLCA, TCA, TUDCA anti	
*Xylanibacter*	Pos.	Pos.	Pos.	Neg.	↑TCDCA, TUDCA pro	
*Parbacteroides*	Neg.	Neg.	Pos.	Pos.	↑ TCA, GCA, TLCA, TDCA pro	↓CA
*Alistipes*	Neg.	Neg.	Pos.	Pos.	↑TCA, GCA, TDCA pro	

*Data taken from (Xie et al., 2016a)

Pos. and Neg. refer to the presence or absence of the bacteria in the feces

#Means that the change in bacteria abundance or BA level either promotes (pro-) or inhibits (anti-) inflammation

There are a few new genera not encountered in the other studies such as *Desulfovibrio*, *Parasutterella*, *Barnsiella* and *Odoribacter*. One can see from these data that organisms that are negatively associated with the disease stages tend to decrease BAs in the liver which cause inflammation while those organisms that are positively associated with the disease stages tend to increase toxic BAs in the liver. There are some discrepancies but this may be due to the necessity for pursuing taxonomy to specific strains or species to identify strains that are more pro-inflammatory. The use of BA profiles can further our understanding of the inflammatory status of the TME in a non-invasive way.

Thus far we have focused mainly on changes in the gut microbiota composition correlated with disease progression and have observed a shift in microbiota composition. It starts with an almost abrupt decrease in anti-inflammatory commensal bacteria followed by increased pro-inflammatory species as one progresses from early virus infection to fibrosis. The progression to cirrhosis seems to be made mainly by fold-changes and the appearance of a few new species in order to further elevate inflammation. As one proceeds to HCC, however, there is shift in the balance between inflammatory and anti-inflammatory genera of bacteria and more anti-inflammatory or immune-modulating species appear to create a new homeostatic condition to preserve the life of the tumor.

## Changes in the Gut Microbiota Associated with CRC

CRC-related microbiota could be classified to three distinct patterns (Mizutani et al., 2020). First, the abundances of gut microbes such as pro-inflammatory bacteria were elevated from stage 0 to more advanced stages, such as *Fusobacterium nucleatum*, *Peptostreptococcus anaerobius*, *Peptostreptococcus stomatis*, and *Parvimonas micra*., *P*. *anaerobius*, *P*. *stomatis* and *P*. *micra* were predominantly enriched in stage I/II and stage III/IV, and their abundances decreased after tumor resection, which implies that these species might not cause carcinogenesis but were adapted to the cancerous environment (Yachida et al., 2019). Second, the abundances of some microbes, such as *Atopobium parvulum* and *Actinomyces odontolyticus*, increased in multiple polypoid adenomas and/or in stage 0 but were not increased in more advanced stages. Third, those anti-inflammatory microbes or some probiotics, such as butyrate producers (i.e., *Lachnospira multipara* and *Eubacterium eligens*) and *Bifidobacterium longum* were depleted with the progression of CRC. Like HCC, there are changes in the composition of the microbiota as the disease progresses through different stages (Liu et al., 2020).

In the first study for discussion of CRC (Table 8), a metagenomics shotgun sequencing of 156 fecal samples was done (Feng et al., 2015). The samples were from 55 HCs, 42 advanced adenoma and 41 carcinoma patients.

**Table 8:** Differential bacteria (fold-changes in relative abundance) in advanced adenoma and carcinoma relative to HC*.

Genus	Shift in relative abundance for advanced adenoma	Anti- or pro- inflammation	Shift in relative abundance for carcinoma	Anti- or pro- inflammation
*Coprococcus*	1-fold↑	Anti-	1-fold↓	Pro-
*Holdemania*	1-fold↓	Pro-	1-fold↑	Anti-
*Bacteroides*	1-fold↑	Anti- or pro-	2-fold↑	Anti- or pro-
*Alistipes*	1-fold↑	Anti-	2-fold↑	Anti-
*Escherichia*	1-fold↓	Pro-	2-fold↑	Pro-
*Parabacteroides*	1-fold↑	Anti-	3-fold↑	Anti-
*Thetaiotaomicron*	1-fold↑	Pro-	2-fold↑	Pro-
*Ruminococcus bacterium*	NC (no change)		2-fold↑	Anti- or pro-
*Pseudoflavonifactor*	1-fold↑	Anti-	2-fold↑	Anti-
*Xylanisolvens*	NC		2-fold↑	Anti-
*Prevotella*	1-fold↑	Anti- or pro-	3-fold↑	Anti- or pro-
*Odoribacter*	NC		2-fold↑	Anti-
*Parvimonas*	NC		1-fold↑	Pro-
*Dialister*	2-fold↑	Pro-	2-fold↑	Pro-
*Bilophila*	NC		2-fold↑	Anti-
*Flavonifactor*	NC		1-fold↑	Anti-
*Oscillibacter*	1-fold↑	Pro-	2-fold↑	Pro-
*Ovatus*	1-fold↑	Pro-	3-fold↑	Pro-
*Barnsiella*	1-fold↑	Anti-	3-fold↑	Anti-
*Methanobrevibacter*	4-fold↑	Pro-	4-fold↑	Pro-
*Citrobacter*	1-fold↓	Anti-	2-fold↑	Pro-
*Fusobacterium*	2-fold↑	Pro-	4-fold↑	Pro-
*Acidaminoccus*	2-fold↓	Pro-	3-fold↑	Anti-
*Porphyromonas*	2-fold↑	Pro-	5-fold↑	Pro-
*Ruminococcus*	NC		1-fold↓	Pro-
*Bifidobacterium*	NC		2-fold↓	Pro-
*Streptococcus*	2-fold↓	Anti- or pro-	2-fold↓	Anti- or pro-
*Difficile*	1-fold↓	Anti-	2-fold↓	Anti-
*Actinomyces*	NC		2-fold↓	Pro-
*Marvinbryantia*	1-fold↑	Anti-	1-fold↓	Pro-

*Data taken SI from (Feng et al., 2015)

Some microbes are associated with CLD and HCC as well as new organisms more specific for CRC. *Thetaiotaomicron*, *Difficile*, *Oscillibacter*, *Ovatus*, *Methanobrevibacter*, and *Porphyromonas* have been reported as opportunistic pathogens, and are implicated in IBD, IBS, ulcerative colitis, and CRC (Saitoh et al., 2002; Kim et al., 2012; Dai et al., 2018; Porter et al., 2018; Sandhu and McBride, 2018; Wu et al., 2019). Some other opportunistic pathogens, such as *Parvimonas* and *Dialister* are implicated with oral infections and liver abscesses (Chaucer et al., 2018; Soeiro et al., 2019). *Coprococcus*, *Holdemania*, *Xylanisolvens*, and *Acidominococcus* have been associated with increased consumption of dietary fiber and are considered as commensal bacteria (Voreades et al., 2014; Despres et al., 2016; Barrett et al., 2018). *Marvinbryantia* is a member of Clostridia and ferments cellulose and methylcellulose. It also boosts the production of succinate (Rey et al., 2010).

What we can conclude from this initial study is that the contribution of the gut microbiota for CRC appears to be relatively pro-inflammatory relative to the adenoma stage although there are anti-inflammatory microbes present. One can also see that there is a different group of gut microbiota from what was seen in HCC that has shifted and thus the TME for CRC, if it receives contributions from the gut microbiota for its modeling, is different from that of HCC based on the idea that the gut microbiota fuels the tumor as well as the host and are a lifeline for the cancer to continue to grow.

A second study (Table 9) was done across all three stages of the disease, polyps, advanced adenoma, and carcinoma (Zhang et al., 2018). The cohort studied included 130 CRC, 88 advanced adenoma, 62 patients with polyps and 130 HCs. What was unique is that all the taxonomy was reported at the genus/species level.

**Table 9:** Fold change relative to HC across the stages of CRC for differential bacterial species*.

Species	Polyps	Anti- or pro- inflammation	Adenoma	Anti- or pro- inflammation	CRC	Anti- or pro- inflammation	Function of bacterial species
*Eubacterium eligens*	1.4↓	Pro-	1.7↓	Pro-	3.7↓	Pro-	Butyrate producing
*Coprococcus comes*	1.4↓	Pro-	1.2↓	Pro-	1.7↓	Pro-	SCFAs
*Eubacterium hadrum*	1.1↓	Pro-	1.2↓	Pro-	2.1↓	Pro-	SCFAs
*Fusicatenibacter saccharivorans*	1.6↓	Pro-	1.3↓	Pro-	1.3↓	Pro-	SCFAs, inhibits UC in mice (Takeshita et al., 2016)
*Blautia faecis*	1.2↓	Pro-	1.1↓	Pro-	1.3↓	Pro-	SCFAs
*Roseburia faecis*	1.5↓	Pro-	1.3↓	Pro-	1.3↓	Pro-	SCFAs
*Eubacterium hallii*	1.2↓	Pro-	NC	Pro-	1.2↓	Pro-	SCFAs
*Ruminococcus lactaris*	1.8↓	Pro-	2.2↓	Pro-	2.1↓	Pro-	Improve metabolic syndrome (Upadhyaya et al., 2016)
*Eubacterium desmolans*	1.1↓	Pro-	1.1↓	Pro-	1.4↓	Pro-	SCFAs
*Clostridium lactatifermentans*	1.3↓		1.3↑		2.5 ↑		?
*Streptococcus salivarius*	1.5↑	Anti-	1.2↑	Anti-	1.5↓	Pro-	Silences innate immune system
*Peptostreptococcus stomatis*	4.6↑	Pro-	1.8↑	Pro-	91↑	Pro-	Opportunistic oral pathogen
*Parvimonas micra*	6↑	Pro-	4 ↑	Pro-	120↑	Pro-	Opportun-istic oral pathogen
*Gemella morbillorum*	3.7↓	Anti-	3.3↓	Anti-	3↑	Pro-	Endodontic pathogen
*Dialister pneumosintes*	2↑	Pro-	NC	Pro-	141↑	Pro-	Oral pathogen
*Porphyromonas asaccharalytica*	1.7↓	Pro-	1.4↑	Pro-	101↑	Pro-	Oral pathogen
*Solobacterium moorei*	2↓	Anti-	3↓	Anti-	4.7↑	Pro-	Oral pathogen
*Eisenbergiella tayi*	1.4↓		2.8 ↑		3.9 ↑		?
*Fusobacterium nucleatum*	2↑	Pro-	3↑	Pro-	170↑	Pro-	Tumor associated
*Ruminococcus torques*	NC		1.4↑	Pro-	2↑	Pro-	Mucolytic, increased in IBD (Png et al., 2010)
*Eggerthella lenta*	1.1↑	Pro-	1.1↑	Pro-	2.4↑	Pro-	Associated with Crohn’s disease (Thota et al., 2011)
*Clostridium symbiosum*	1.3↑	Pro-	1.8↑	Pro-	3.4↑	Pro-	Promising biomarker for CRC(Xie et al., 2017)
*Campylobacter rectus*	NC		NC		5↑	Pro-	Oral pathogen
*Clostridium scindens*	2.5↑	Pro-	3.2↑	Pro-	3.5↑	Pro-	Produces secondary Bas (Greathouse et al., 2015)

*Data taken from (Zhang et al., 2018)

The results in Table 9 demonstrate just how different conclusions can be made if taxonomy is taken to the species level. There is a clear increase in inflammation across the stages of the disease with little abatement at the tumor stage as was seen in all the other studies that were discussed.

The final summary table (Table 10) is a comparison of gut microbiota shifts between CRC and HCC. The three types of HCC, HCV, HBV and non-viral gut microbiota shifts are listed separately. It is immediately apparent that there are many more bacteria genera reported for CRC than for HCC. The table also indicates that there are definite differences in gut microbiota shifts for all three types of HCC and that there are also common organisms reported for both CRC and HCC. Some increasing and decreasing trends are also sometime opposite for the different cancer types. The emerging picture is that CRC is distinct from HCC in their respective, gut microbiota driven progression and maintenance. It cannot, however be excluded that there are some functional redundancies amongst the different microbiota associated with the two cancers.

**Table 10:** Summary table comparing gut microbiota shifts for HCV, HBV and non-viral HCC to CRC*. Genus level taxonomy is presented.

HCV-HCC (↑)	HCV-HCC (↓)	HBV-HCC (↑)	HBV-HCC (↓)	NV-HCC (↑)	NV-HCC (↓)	CRC (↑)	CRC (↓)
*Acinetobacter*							
*Prevotella*			*Prevotella*		*Prevotella*	*Prevotella*	
*Veillonella*		*Veillonella*		*Veillonella*			
*Phascolarctobacterium*					*Phascolarctobacterium*		
*Faecalibacterium*			*Faecalibacterium*		*Faecalibacterium*		
*Streptococcus*		*Streptococus*					*Streptococcus*
	*Blautia*		*Blautia*				*Blautia*
*Parabacteroides*		*Parabacteroides*		*Parabacteroides*		*Parabacteroides*	
*Bifidobacterium*			*Bifidobacterium*	*Bifidobacterium*			*Bifidobacterium*
	*Bacteroides*	*Bacteroides*			*Bacteroides*	*Bacteroides*	
		*Dorea*					*Roseburia*
			*Roseburia*	*Roseburia*			
			*Coprococcus*				*Coprococcus*
			*Clostridium*			*Clostridium*	
			*Eubacterium*				*Eubacterium*
			*Klebsiella*				
			*Haemophilus*				
	*Ruminococcus*						*Ruminococcus*
	*Lachnospiraceae*						
		*Escherichia*					
*Shigella*		*Escherichia*					
*Shigella*		*Escherichia*					
*Shigella*							
			*Enterococcus*	*Enterococcus*			
				*Proteus*			
	*Fusobacterium*				*Fusobacterium*	*Fusobacterium*	
*Ruminiclostridium*					*Ruminiclostridium*		
					*Lachnoclostridium*		
				*Subdoligranulum*			
					*Pseudobutyrivibro*		
		*Alloprevotella*					
				*Alistipes*		*Alistipes*	
						*Holdemania* *Thetaiomicron* *Pseudoflavonifactor* *Xylamisolvens* *Odoribacter* *Parvimonas* *Dialister* *Bilophila* *Flavonifactor* *Oscillobacter* *Ovatus* *Barnsiella* *Methanobrevibacter* *Citrobacter* *Acidaminococcus* *Porphyromonas* *Peptostreptococcus* *Gemella* *Solobacterium* *Eisenbergiella*	*Difficile* *Actinomyces* *Marvinbryantia* *Fasicatenibater*

*Data compiled from Tables 3, 5, 6, 8, 9 presented in this review

## Conclusion

In this review we sought to discover if the tumor microenvironment was similar for the various types of HCC and to compare HCC vs. CRC. If we use the idea that the microbiota co-metabolizes with the tumor in the same way as it provides for the host, then we can think of it as a lifeline for the tumor. Targeting the lifeline of a tumor is not a new concept as one of the first attempts to do this was with anti-angiogenic drugs with the notion of “starving the tumor” by denying it a blood supply. Perhaps we can manipulate the microbiota to achieve a similar effect. If the gut microbiota do contribute to the modelling of the TME via providing nutrients or immune modulation then from the data presented here, HCC and CRC have a very different TME. In addition, it can be seen from the Table 10 that HCC spawned from three different etiologies may also have distinct differences in their TME. Although we just chose just a few isolated studies to discuss, certain consistencies did reveal themselves. For instance, the overgrowth of *Streptococcus* that was more dominant for HCV-HCC relative to HBV-HCC. There was also a slightly more pro-inflammatory profile for non-viral HCC when compared to viral HCC and perhaps this is because non-viral HCC begins on a background of an already “homeostatic” inflamed intestine due to the shift in the balance which happened to cause IBD or metabolic syndrome. It must be emphasized that more studies addressing evolutionary changes in the gut microbiota correlated to disease etiology and/or stage need to be done. It should also be cautioned that some results may differ, especially between different labs as a result of different sampling techniques, depth of sequencing and patient populations. However, the ability to manipulate the gut microbiota to kill tumors is certainly something worth reaching for.

The limitation of the review is that fecal microbiota composition is not equivalent to the concept of “TME”. The microbiome profile of tumor tissue would be more appropriate than fecal microbiome. In a study conducted by Sung and Yu groups, gut mucosal microbiome of adenoma and adenoma-adjacent mucosae, carcinoma and carcinoma-adjacent mucosae have been analyzed. The results suggest that a taxonomically defined microbial consortium, such as *Bacteroides fragilis*, *Gemella*, *Parvimonas*, *Peptostreptococcus*, and *Granulicatella* are implicated in the development of CRC (Nakatsu et al., 2015). Another study on CRC also found that the colorectal mucosal microbiota were changed with the progression of CRC, with an increasing trend in the abundances of Bacteroidetes, Firmicutes and Fusobacteria, and a decreasing trend in the abundance of Proteobacteria from stage I to IV (Pan et al., 2020). As to liver diseases, the alcohol-induced liver disease mouse models showed that bacterial translocation to the liver might be associated with microbiota changes in the distal gastrointestinal tract (Bluemel et al., 2019).
